# Supplementary material for: MetaRibo-Seq measures translation in microbiomes
Source: Nat Commun. 2020 Jun 29;11:3268. doi: 10.1038/s41467-020-17081-z (PMC7324362; doi:10.1038/s41467-020-17081-z)
Supplement: Supplementary file 10 — Supplementary Data 7 [file 41467_2020_17081_MOESM10_ESM.zip › File2/Confidence_High_Taxonomy/277140_out.krona.html]

Javascript must be enabled to view this page.

members
magnitude
magnitudeUnassigned
count
unassigned
taxon
rank

277140\_out

24

superkingdom
2
24

24
976
phylum

class
200643
24

order
24
171549

815
24
family

24
1

SRS147653\_contig\_number\_contig-100\_561.55892
816
genus

1
457391
species

SRS043001\_contig\_number\_7182

species

SRS014613\_contig\_number\_14247SRS048060\_contig\_number\_5394SRS064276\_contig\_number\_15720SRS1041145\_contig\_number\_contig-100\_94.184167SRS1055043\_contig\_number\_26466SRS1055067\_contig\_number\_12208SRS1055099\_contig\_number\_6888
7
2292935


SRS022524\_contig\_number\_contig-100\_753.86772SRS143598\_contig\_number\_12465
species
457395
2

821
4

SRS016018\_contig\_number\_14154SRS019685\_contig\_number\_contig-100\_671.221969SRS064232\_contig\_number\_3634SRS101433\_contig\_number\_15781
species


SRS013800\_contig\_number\_16016SRS014923\_contig\_number\_contig-100\_1202.238590SRS018049\_contig\_number\_777SRS019286\_contig\_number\_5276SRS024435\_contig\_number\_33477SRS052078\_contig\_number\_2770SRS098655\_contig\_number\_5737SRS101268\_contig\_number\_500SRS893270\_contig\_number\_23277
species
357276
9
